# The moderating role of specific self-efficacy in the impact of positive mood on cognitive performance

**DOI:** 10.1007/s11031-014-9469-3

**Published:** 2015-01-22

**Authors:** Tomasz Niemiec, Kinga Lachowicz-Tabaczek

**Affiliations:** Institute of Psychology, University of Wroclaw, ul. Dawida 1, 50-527 Wrocław, Poland

**Keywords:** Mood, Specific self-efficacy, Cognitive performance

## Abstract

Research concerning the impact of positive mood on cognitive performance is inconsistent. We suggest that specific self-efficacy moderates this relationship. The current study proposed that participants in a positive mood with a high level of specific self-efficacy would anticipate mood-maintaining success on a task. Hence, they would be more strongly motivated, and perform better on the task, than individuals in other moods. Conversely, participants in a positive mood with low specific self-efficacy should expect mood-threatening failure. Thus, these individuals should be less motivated and perform more poorly than individuals in other moods. The current study included 139 participants with different levels of specific self-efficacy performing a comprehension task in either a positive or negative mood or a control condition. Results confirmed our hypothesis whereby specific self-efficacy affects cognitive performance but only during a positive mood. These findings support the role of specific self-efficacy in maintaining positive mood by regulating task activity.

## Introduction

Studies investigating cognitive performance during a positive mood have yielded inconclusive results. Several studies have shown that positive mood impairs cognitive performance (Abele 1992; Mackie and Worth 1989; Schwarz and Clore 1983), while others suggest the opposite pattern (Bohn-Gettler and Rapp 2011; Isen et al. 1987), when compared to negative mood. To resolve these inconsistencies, models of potential moderators have been suggested (Bless et al. 1996; Martin et al. 1993; Wegener et al. 1995, Ziegler 2014). These moderating models propose that a positive mood leads to either better or worse cognitive performance depending on situational circumstances (Bless et al. 1996) or regulatory aspects of the positive mood itself (Martin et al. 1993; Wegener et al. 1995; Ziegler 2014).

In a model proposed by Bless et al. (1996), situational demands moderate the impact of a positive mood on cognitive performance. Individuals in a positive mood usually do not efficiently process information because they rely on general knowledge such as scripts and stereotypes. However, this does not imply that individuals in a positive mood are unmotivated or inefficiently process information in general. They immediately begin efficient processing when the situation demands it (i.e., when general knowledge is not applicable or when the task is complex).

Martin et al. (1993) proposed a model in which the moderator is related to regulatory aspects of a positive mood. According to this *mood*-*as*-*input* model, individuals can interpret a positive mood differently at both the beginning and during a task. They might interpret a positive mood as a sign that they have already performed well on the task. This interpretation could lead to a withdrawal of effort and decreased performance. However, individuals might also regard their positive mood as a sign that they are enjoying the current task. As such, individuals will put more effort into the task and improve their performance.

Ziegler (2014) also claims that a mood at the beginning and during a task might be crucial for processing, although not as a signal related to formal processing parameters (e.g., putting in or withdrawing effort as in Martin et al.’s account) but as a factor interacting with processing content. Ziegler claims that individuals create expectancies regarding processing content based on their mood. If the information is incongruent with these expectancies, it might be taken as surprising and processed better. For example, during a positive mood, individuals expect pleasant information. If an unpleasant one comes on, it is incongruent with their mood-based expectancies. Thus, it is taken as more surprising and processed better than a pleasant one (unless it impairs the goal of affective regulation).

Wegener and Petty’s *hedonic contingency theory* (Wegener and Petty 1994; Wegener et al. 1995) involves an even more complex model of moderator related to regulatory aspects of a positive mood. Unlike Martin et al.’s (1993) and Ziegler’s (2014) models, Wegener and Petty assert that individuals’ performance depends on the mood not only at input (i.e., at the beginning or during processing) but also at output (i.e., at the end or after processing). Wegener and Petty assumed that when individuals are in a positive mood, they effectively process only the material that is relevant to their mood and aids with mood maintenance. Thus, when in a positive mood, individuals endorse a strategic approach to information processing and effectively process only information with desirable mood consequences. Conversely, information is processed less effectively when threatening to a positive mood (Wegener et al. 1995).

### The role of specific self-efficacy (SSE)

The current study is based on a model similar to *hedonic contingency theory*. As in this approach, we assume that individuals in a positive mood strive to maintain this mood. However, we suggest that factors other than processing pleasant information also serve this purpose. We hypothesized that individuals maintain their positive mood by engaging in a task where they feel *efficacious*. During such a task, they anticipate success, which could maintain their positive mood. Therefore, these individuals will be strongly motivated to perform such a task and achieve better results. Conversely, if individuals feel inefficient during a particular task, they might anticipate failure, and this could dampen their positive mood. These individuals might be less motivated to perform such tasks when in a positive mood and could perform worse.

Thus, we predict that *specific self*-*efficacy* (SSE) might moderate the relationship between positive mood and cognitive performance. SSE in this context is regarded as an individuals’ belief that they can mobilize motivation, cognitive resources, and courses of action to meet situational demands (Wood and Bandura 1989). Thus, unlike the aforementioned models, SSE works like a trait-based characteristic (Gupta et al. 2013; Low et al. 2005), influencing the relationship between mood and performance. In the models described previously, moderators were temporary circumstances: situational demands in Bless et al.’s (1996) proposition, current interpretation of the meaning of a positive mood in Martin et al.’s (1993) model, congruency of ongoing information with mood-based expectations in Ziegler 2014 approach, and hedonic relevance of information to the current mood in Wegener and Petty’s (Wegener et al. 1995) model.

To test our hypothesis, we conducted a study with tree groups. Two groups received either a positive or negative mood induction. We also included a control group in which no particular mood was induced.

We predicted that individuals in a positive mood with high SSE would anticipate success during a relevant activity. Here, success can serve to maintain their positive mood. Therefore, individuals with high SSE while in a positive mood should be more strongly motivated, and perform better, than individuals with high SSE performing the task in other mood conditions.

Conversely, individuals in a positive mood with low SSE should anticipate failure during a relevant activity. Failure could impair their mood, leading to less motivation and poorer performance compared to individuals with low SSE level performing the task in other mood conditions.

The expectancies of diminished motivation among individuals with low SSE and enhanced motivation of high SSE ones are based on Bandura’s work (Bandura 1977). As high SSE was confirmed to be related to the anticipation of success, low SSE was proved to be related to a fear of failure, avoidance motivation, and avoidance coping responses (Bandura 1986; Bartels 2007; Li and Yang 2009). Alter and Forgas’ (2007) findings may suggest that such tendencies concerning low SSE may be even more pronounced during a positive mood. The authors made their participants doubt their abilities and found that participants engaged in auto-handicapping behavior while in a positive mood as compared to those in neutral and negative moods. According to the authors, this strategy was used in order to protect participants’ mood against the consequences of failure.

Based on assumptions regarding expectancies of low versus high SSE individuals in a positive mood, we expected that the effect of SSE on mood and cognitive performance should be more pronounced for positive relative to the other mood groups. Specifically, cognitive performance among individuals with high SSE should be enhanced while performance for those with low SSE should be impaired.

We also predicted that cognitive performance among individuals in the negative mood and control conditions would be less affected by SSE than performance of individuals in the positive mood condition. This is because individuals in negative or neutral moods are experiencing a different situation from participants in a positive mood. In comparison to the positive mood participants, individuals in the negative or neutral moods should find more tasks rewarding. When these individuals engage in whatsoever activity, they might experience an improved mood. When participants in a positive mood engage in the same activity, it is more likely to deteriorate their pleasant mood. Therefore, according to Wegener and Petty’s model, individuals in the negative and neutral mood conditions might not even analyze the ongoing activity’s consequences to their mood as compared to the positive mood participants (Wegener and Petty 1994; Wegener et al. 1995). Hence, motivation among individuals in negative and neutral moods should be less dependent on whether the task is satisfying and whether they feel efficacious. Accordingly, these participants’ cognitive performance should be less dependent on SSE.

## Methods

### Participants

Participants were 67 students from the College of Management “Edukacja” and 72 students from the University of Wrocław, for a total of 139 students. This included 58 men (42 %) and 81 women, aged 19–50 years (*M* = 24.44; *SD* = 6.88). All participants volunteered and were not paid for their participation.

### Procedure

The study was carried out in various groups of 10–20 participants. Each group was randomly assigned to one of three conditions: positive mood, negative mood, and control condition. The study included just one session lasting approximately 45 min. In the two experimental conditions (positive *vs.* negative mood), participants were informed that they were taking part in two studies. The first study was introduced as examining perception of film clips and the second study as research on reading comprehension. Two experimenters conveyed the impression that there were indeed two studies. In fact, both studies constituted one experiment, with the first part inducing the specific mood and the second part measuring SSE, mood, and performance. The purpose of this design was to obscure the true aims of the study, which was to examine the impact of mood on task performance. Previous experiments assessing the influence of mood on cognitive performance employed a similar procedure (Bless et al. 1996; Martin et al. 1993; Wegener et al. 1995), with the mood induction as an independent task. There was no mood manipulation in the control condition, but other variables were measured in the same order as in the experimental groups (i.e., SSE, mood measure, and reading comprehension performance).

#### Mood induction

In the experimental conditions, the first experimenter told participants that his study explored perception of film clips. The experimenter then presented 7-min films. In the positive mood condition, the film showed a French comedic performance recorded with a hidden camera. In the negative mood condition, the film presented the hunger problem in Africa. A pilot study was conducted to ensure that these films actually induced the intended moods. Results of this pilot study (*N* = 74) confirmed mood manipulation effectiveness. Participants watching the positive mood-inducing film experienced a significantly higher positive mood than did participants watching the negative mood-inducing film, *F*(2, 71) = 10.25; *p* = .002; η^2^ = .13. Next, participants completed a buffer scale designed to convince them that the study indeed explored film perception. The scale included such statements as “When watching the film, I was identifying myself with one of the main characters” and “I watch films like this frequently.” The participants were asked to rate each statement on a four-point scale from 1 (“I definitely agree”) to 4 (“I definitely disagree”). Afterwards, the first experimenter thanked participants for their participation. He then left the room and the other experimenter entered, who told participants that the second study would assess reading comprehension. As there was no mood manipulation for the control group, control participants only performed the second part of the study.

#### Specific self-efficacy (SSE) questionnaire

A questionnaire assessing SSE related to reading comprehension was created specifically for the present study. It was based on the assumption that SSE consists of participants’ beliefs that they could mobilize cognitive resources, motivation, and courses of action needed to meet situational demands (Wood and Bandura 1989). Thus, the questionnaire contained four statements referring to participants’ ability to mobilize resources toward accomplishing the reading comprehension tasks (“Despite putting in the effort, I am often not good at reading comprehension.” [reversed], “Reading comprehension comes easy to me,” “My ability to read with understanding is at a satisfactory level,” “If I really try, I can read and comprehend material very well”). Participants had to rate to what extent they related to these statements using a six-point scale from 1 (“I definitely disagree”) to 6 (“I definitely agree”); Cronbach’s α = .77; average inter-correlation *r* = .46. A pilot study revealed that results of this questionnaire correlated with reading comprehension task performance (*r* = .30; *p* < .001). These results match those observed in other studies (Chen et al. 2000; Gupta et al. 2013).

#### Mood-manipulation checks

After completing the SSE scale, participants rated their mood on a scale used in previous studies (Lachowicz-Tabaczek and Śniecińska 2014). The goal was to assess mood manipulation effects. The scale was similar to those implemented by other researchers (Bless et al. 1996; Martin et al. 1993; Wegener et al. 1995). Similarly, like in Martin et al. experiment, the questionnaire contained 13 mood states (e.g., worried, amused, happy, relaxed, tensed, depressed, etc.), a greater number than was assessed in Bless’ et al. and Wegener’s and Petty’s studies. Participants were asked to rate how much they felt each adjective on a 9-point scale from 1 (“not at all”) to 9 (“fully”), as in Bless’ et al. and Wegener’s and Petty’s experiments. Answers for negative mood items were summed and subtracted from the positive mood items to get a total mood score (Cronbach’s α = 0.84). Higher scores indicated a more positive mood.

#### Cognitive task

Participants next performed the reading comprehension task to assess cognitive performance. Participants first read a text containing about 4,500 characters addressing issues related to environmental protection. After reading the text, participants answered 16 multiple-choice questions. Each question had four possible answers. For each item where participants chose the correct answers and omitted all incorrect answers, participants received 4 points, resulting in a maximum of 64 points (i.e., a total of four points per item). Participants were fully debriefed afterwards.

## Results

### Mood-manipulation checks

The manipulation was effective. We conducted a one-way analysis of variance (ANOVA) with the three experimental conditions (positive mood, negative mood, control) as the independent variable and mood as the dependent variable. Results revealed a significant main effect of mood condition in the expected direction, *F*(2, 136) = 17.99, *p* < .001, η^2^ = .21. Planned comparisons revealed that participants in the positive mood group rated their mood as significantly higher (*M* = 26.25), *F*(2, 136) = 6.74, *p* = .01, η^2^ = .05, while participants in the negative mood group rated their mood as significantly lower (*M* = 7.08), *F*(2, 136) = 10.51, *p* = .002, η^2^ = .07, than participants in the control group (*M* = 18.26).

Because the SSE measure was administered after the mood manipulation, we had to make sure that the mood manipulation did not influence SSE scale results. Thus, we conducted another one-way ANOVA in which the three experimental conditions were again the independent variable, while the dependent variable was SSE scale results. There was no main effect of experimental condition, *F*(2, 136) = 1.2, *ns*, η^2^ = .02. Planned comparisons also revealed no differences between groups regarding SSE: positive mood group (*M* = 18.2) versus negative (*M* = 17.16), *F*(2, 136) = 2.39, *ns*, η^2^ = .02, and control group (*M* = 17.76), *F*(2, 136) = .46, *ns*, η^2^ = .003.

To check also the independence of SSE and mood results, we calculated the correlation between these two variables, which turned out to be non-significant (*r* = .05, *ns*). We then conducted a regression analysis to examine the effect of SSE in interaction with mood manipulation on mood results. Two contrast-coded variables were created. The first contrast was C_1_; this contrast compared the positive mood group (coded as “2/3”) with the negative mood and control groups (each coded as “−1/3”). The second contrast was C_2_, which compared the negative mood with the control group (negative mood group coded as “−1/2”; control group coded as “1/2”; positive mood group coded as “0”) (Cohen et al. 2003). The independent variables were SSE (mean-centered), experimental condition (in two contrast-coded variables), and interactions between SSE and experimental conditions. The dependent variable was mood score. Results revealed that neither the interaction between C_1_ and SSE (*B* = .48, *SE* = .84, *t*(133) = .57, *ns*) nor the interaction between C_2_ and SSE (*B* = .19, *SE* = 1.18, *t*(133) = .16, *ns*) influenced mood results.

### Cognitive performance

A regression analysis was conducted to verify our hypotheses concerning the moderating role of SSE in mood effects on task performance. Contrast coding was used to code all conditions. The experimental conditions were transformed into two contrast-coded variables. The first contrast was C_1_, which compared the positive mood group (coded as “2/3”) with the negative mood and control groups (each coded as “−1/3”). The second contrast was C_2_, which compared the negative mood with the control group (negative mood group coded as “−1/2”; control group coded as “1/2”; positive mood group coded as “0”) (Cohen et al. 2003). In each case, SSE (mean-centered) and experimental conditions were entered in Step 1, while interactions between these variables were entered in Step 2. The dependent variable was cognitive performance score (i.e., reading comprehension). The regression results are presented in Table 1.

**Table 1:** Regression analysis of mood and specific self-efficacy (SSE) on cognitive task performance (reading comprehension)

Variable	*B*	*SE*
*Step 1*		
Contrast 1: positive mood versus negative mood and control groups	−.16	1.16
Contrast 2: negative mood versus control group	.98	1.5
SSE	.65*	.18*
*Step 2*		
Contrast 1	−.16	1.14
Contrast 2	.64	1.49
SSE	.50*	.19*
Contrast 1 × SSE	.88*	.36*
Contrast 2 × SSE	−.24	.50

*SSE* specific self-efficacy

* *p* < .02

The Step 1 analysis showed a significant main effect of SSE (*B* = .65, *SE* = .18, *t*(135) = 3.68, *p* < .001) but no significant main effects based on coded variables: C_1_ (*B* = −.16, *SE* = 1.16, *t*(135) = −.14; *ns*) and C_2_ (*B* = .98, *SE* = 1.5, *t*(135) = .65; *ns*). The Step 2 analysis revealed a significant interaction between SSE and C_1_ (*B* = .88, *SE* = .36, *t*(133) = 2.47, *p* < .02) but no interaction between SSE and C_2_ (*B* = −.24, *SE* = .5, *t*(133) = −.48; *ns*).

Simple slopes tests showed that, as hypothesized, SSE was a positive predictor of cognitive performance only for participants who were in a positive mood; higher SSE predicted enhanced cognitive performance (*B* = 1.08, *SE* = .25, *t*(133) = 4.36, *p* < .001). SSE did not affect the other groups’ cognitive performance (negative mood group, *B* = .09, *SE* = .38, *t*(133) = .24, *ns*; control group, *B* = .33, *SE* = .32, *t*(133) = 1.02, *ns*).

**Fig. 1:**
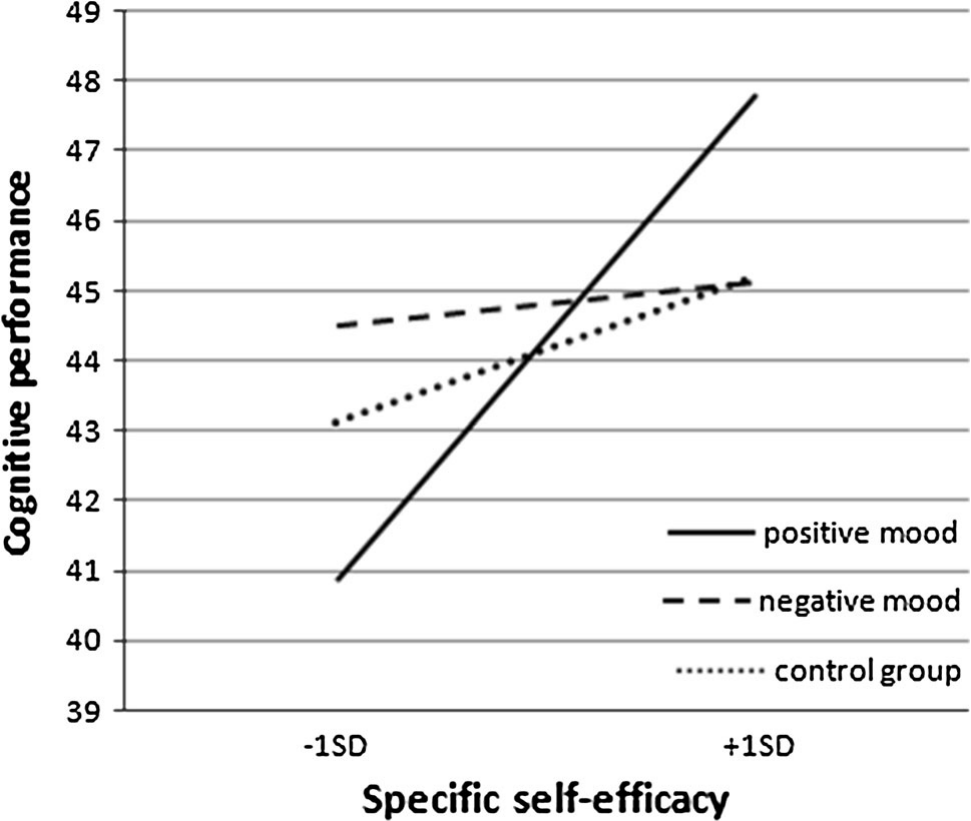
Cognitive task performance as a function of experimental condition and specific self-efficacy (SSE)

Figure 1 illustrates the effects of experimental condition on cognitive performance among the higher (+1 *SD*) and lower (−1 *SD*) SSE individuals. Cognitive performance among higher SSE individuals (+1 *SD*) was significantly better in the positive mood condition than in the other two conditions (*B* = .88, *SE* = .36, *t*(133) = 2.47, *p* < .02). Lower SSE individuals (−1 *SD*) had lower cognitive performance in the positive mood condition than in the other two conditions (*B* = .88, *SE* = .35, *t*(133) = 2.47, *p* < .02).

## Discussion

The current results support our hypothesis that SSE moderates cognitive performance among individuals in a positive mood. Participants with high SSE experiencing a positive mood achieved the highest cognitive performance among all groups. Because of a high SSE, these participants likely anticipated success on the task related to their SSE. As success can maintain their positive mood, these individuals became more motivated to perform, even more so than others with high SSE, which leads them to outperform other high SSE individuals.

Conversely, individuals with low SSE are more likely to anticipate failure during activities from their SSE area. To maintain a positive mood, these individuals might adopt a strategy that protects against failure such as avoiding engagement in the task performance. This could explain why low SSE individuals in a positive mood performed worse on the task as compared to low SSE participants in other moods. As a result, individuals in a positive mood with a low SSE level obtained the lowest cognitive performance among all groups.

As individuals in a positive mood performed best when SSE was high and worst when low, SSE appears to be a valid moderator affecting cognitive performance when in a positive mood. In contrast to previously described moderators, suggesting more temporary influencers (Bless et al. 1996; Martin et al. 1993; Wegener et al. 1995; Ziegler 2014), SSE appears to be more of a trait-based factor influencing the relationship between mood and cognitive performance (Gupta et al. 2013; Low et al. 2005).

### Alternative interpretations: The hedonic contingency approach and performance and enjoyment-based stop rules

One possible alternative explanation for different levels of performance going with different levels of SSE in positive mood might be that SSE changed the experience of the task. For instance, participants high in SSE might have liked the task more than participants low in SSE. This alternative would be more in keeping with Wegener and Petty’s hedonic contingency theory (Wegener et al. 1995), which states that people in a positive mood do better on tasks that they find enjoyable but worse on tasks they find unpleasant. If so, task liking should function as a mediator of the interactive effect of SSE and mood on performance (i.e., in a positive mood, higher SSE should lead to higher task liking which, in turn, should increase performance). To assess this possibility, we conducted a follow-up study that included a 4-item scale measuring the liking of reading comprehension tasks (e.g., “I like reading comprehension tasks,” with responses ranging from 1 “I definitely disagree” to 8 “I definitely agree”). Apart from that, in this study we measured the same variables (with mood questionnaire shortened to 4 items and reading comprehension test shortened to 6 questions) according to the same procedure as our main experiment. One hundred twenty-seven participants took part in this study. First we calculated the correlation between SSE and task liking, which turned out to be moderate and significant (*r* = .66, *p* < .001). Then, we repeated the main analyses from our previous study. The interaction between SSE (mean-centered) and positive mood (calculated as a contrast comparing positive mood to two other conditions; coded: “2/3” as positive mood and “−1/3” as two other groups) was significant (*B* = .21, *SE* = .09, *t*(121) = 2.43, *p* < .02), which replicated our previous results. Meanwhile, when liking (mean-centered) was placed into the regression equation, the interaction between positive mood and performance remained significant (*B* = .21, *SE* = .09, *t*(120) = 2.39, *p* < .02), and inclusion of the liking variable into the regression equation did not increase predictive power of the model (*R* ^2^ *Change* < .001, *F*(6, 120) *Change* = .09, *ns*). This result proves that task liking did not mediate the effect of an interaction between positive mood and SSE on task performance. Thus, an alternative interpretation based on hedonic contingency theory was not supported.

Actually, such findings refer also to alternative interpretation basing on Martin and colleagues’ account (Martin et al. 1993), suggesting that when in a positive mood, low SSE participants might doubt their ability to perform well. In effect, they might pursue a performance-based stop rule using positive mood as a performance indicator. Conversely, high SSE individuals might like the task more and apply an enjoyment-based stop rule. Therefore, when in a positive mood, one should observe task liking mediating the interaction effect between positive mood and SSE on performance (an enjoyment-based stop rule). As described above, the results of our follow-up study did not confirm such an effect. As an enjoyment-based stop rule turned out to be less likely for explaining the obtained results, we cannot relate to the prediction coming from the performance based stop rule postulated in Martin et al.’s model. This is because we had no adequate measure for examining performance concerns and its possible meditational influence on task results. Thus, to fully assess whether Martin and colleagues’ model provides a possible alternative interpretation for the present results, a “performance concern” measure should be included in future research.

Concerning participants in the control and negative mood groups, our analyses revealed they performed at the same level regardless of SSE. Wegener and Petty’s hedonic contingency theory (Wegener and Petty 1994; Wegener et al. 1995) provides a valid explanation for these findings. The authors state that when participants in a positive mood engage in whatsoever activity, their current mood is more likely to decrease since the mood can go nowhere but down. In turn, when individuals in a negative or neutral mood engage in that same activity, they would likely improve or maintain their current mood. In other words, engaging in whatsoever activity is most rewarding for participants who are initially in a negative or neutral mood. Therefore, those individuals might not even analyze the consequences which an ongoing activity brings to their mood. Thus, these participants likely would not base task motivation on whether they found the current task satisfying and whether they will felt efficient performing it. For this reason, SSE probably did not influence cognitive performance among the control and negative mood groups.

### Mood as a resource approach

The present results might also refer to interpretations considering positive mood as a resource. Positive mood can be beneficial for performance on activities related to social interactions, health (Forgas 2006), creativity (Isen et al. 1987), and reading comprehension (Bohn-Gettler and Rapp 2011). However, the current findings somewhat contradict the general idea that positive mood acts as a universally positive resource. A similar conclusion came from Trope et al. (2001) conclusions. Our results suggest that individuals in a positive mood might be less motivated to perform tasks when experiencing low SSE. Thus, they might try to avoid such tasks and would not benefit from a positive mood as a resource. In addition, diminished performance can further decrease these individuals’ SSE. A continuous feedback loop develops, resulting in recurrent decreases in SSE. Intrinsic motivation might also suffer, as SSE is related to a need for competence, which is a basic component of intrinsic motivation (Deci and Ryan 2008). One method for ceasing this negative feedback loop lies within SSE. For instance, high SSE might encourage individuals to engage in specific activities when in a positive mood. Performing the activity when in a positive mood might improve outcomes, which could lead to additional SSE enhancements.

### Limitations and future directions

The generalization that individuals in a positive mood base their motivation on SSE might be subject to additional limitations. The first limitation is related to the importance participants placed on the activity. If the task is not important, success or failure might not modify one’s mood (Frijda 1988). Therefore, SSE might not affect motivation or performance even when individuals are in a positive mood.

Furthermore, even when a certain activity is important for the subject, for implementing a postulated mechanism (i.e., basing motivation on SSE level in positive mood to maintain that mood), individuals must possess some mood regulation experience and know the activities in which they are proficient. Therefore, young or less emotionally intelligent individuals might not show the same moderating effect of SSE, as their mood regulation ability is limited (Chapman and Hayslip 2006).

Even when individuals have the ability to attain and maintain a positive mood, in some cases they might not apply it. Research suggests that there are certain activities for which a neutral mood seems to be most beneficial (Erber and Tesser 1992). Moreover, there are situational (Stearns and Parrott 2012) and trait like factors (Tamir 2005) that might determine when individuals prefer being in a negative mood.

We propose further experiments in which SSE could be more directly manipulated. This would allow for more justified causal explanations regarding SSE and its modulatory effect on the relationship between positive mood and cognitive performance.

It is also important to obtain insight into the mechanism(s) responsible for determining how a positive mood affects cognitive performance. We postulate that this mechanism likely refers to enhanced motivation among high SSE individuals and diminished motivation among low SSE individuals. These motivational factors could be assessed by measuring cognitive phenomena, such as task success value and task importance, while also examining certain behavioral tendencies (e.g., auto-handicapping).

Future research should also examine support for the notion that motivation based on SSE indeed maintains a positive mood. This can be accomplished via a final mood measure at the end of a research session, after cognitive performance has been assessed.

Furthermore, apart from SSE, there might be additional trait-like moderators of cognitive performance while in a positive mood. For instance, neuroticism might be a plausible moderator in that highly neurotic individuals limit their information processing while in a positive mood in order to avoid being exposed to worrisome material. Thus, further explorations regarding trait-like moderators of the impact of mood on cognitive performance are promising perspectives.
